# Rhizosheath Characteristics of Annual Ephemerals in Sandy and Gravelly Habitats of Northern Xinjiang Deserts

**DOI:** 10.1002/ece3.72324

**Published:** 2025-10-25

**Authors:** Yu Ding, Cheng Lv, Kangwei Jiang, Xinyu Xia, Zhiqing Zhang, Qingqing Zhang

**Affiliations:** ^1^ College of Life Science Xinjiang Agricultural University Urumqi Xinjiang China; ^2^ Xinjiang Agricultural University Xinjiang Key Laboratory of Biological Ecological Adaptation and Evolution in Extreme Environments Urumqi Xinjiang China; ^3^ College of Grassland Science Xinjiang Agricultural University Urumqi Xinjiang China; ^4^ College of Resources and Environment Xinjiang Agricultural University Urumqi Xinjiang China

**Keywords:** annual ephemerals, interspecific variations, morphological characteristics of the rhizosheath

## Abstract

The rhizosheath is an important root trait that plays a critical role in enhancing plant uptake of water and nutrients, contributing to the adaptation of plant species to specific environmental conditions. This study investigates the formation and characteristics of rhizosheaths in annual ephemeral plants in the desert environment of northern Xinjiang through systematic field surveys and comparative analyses. Key findings include: (1) A total of 67 annual ephemeral species were surveyed, with 15 species exhibiting distinct rhizosheath structures, predominantly from the Poaceae family. (2) Among the rhizosheath‐forming species, 11 were identified in sandy habitats, while 7 were found in gravelly habitats. Notably, three species (*Eremopyrum distans*, *Eremopyrum triticeum* , and *Eremopyrum orientale)* were observed to form rhizosheaths in both habitat types. Rhizosheath traits, such as length, diameter, surface area, volume, relative weight, and development index, were significantly greater in sandy environments than in gravelly ones. These results highlight the species‐specific characteristics of rhizosheath formation among desert annual ephemerals in northern Xinjiang and demonstrate that sandy habitats are more favorable for rhizosheath development. This study provides valuable insights into the ecological strategies of desert plants and contributes to our understanding of their adaptive mechanisms in arid environments.

## Graphical abstract

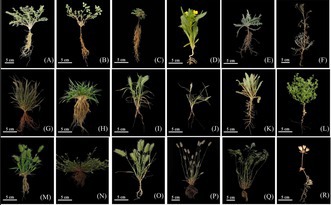

## 1 Introduction

The rhizosheath refers to the soil that adheres to plant roots, facilitated by mucilages produced by both plants and microorganisms, which enhance soil aggregation and lead to the formation of a protective sheath around the root surface (Ma et al. 2011; Brown et al. 2017). This rhizosheath performs essential physiological and ecological functions (Hallett et al. 2022), enabling plants to cope better with abiotic stresses such as drought and nutrient deficiency (Delhaize et al. 2012; Paez‐Garcia et al. 2015; Brown et al. 2017; Basirat et al. 2019). Consequently, the rhizosheath is considered a crucial adaptive trait that helps plants mitigate the effects of prolonged drought and other stresses (Hallett et al. 2022). The important ecological value of rhizosheath is of great significance for understanding the mechanisms of plant growth, development, and adaptation to the environment; the abundant biological resources in rhizosheath play an important role in understanding the formation, development, and function of rhizosheath, and revealing the interaction mechanism between plants and microorganisms. Under the background of future climate change, limited resources, and global economic growth, it has important theoretical and practical value for the interaction between plants and soil ecosystems, promoting sustainable agricultural development, improving the stress resistance and productivity of crops, and providing a scientific basis and technical support for agricultural production and ecological restoration.

The rhizosheath is commonly found in numerous angiosperm species, particularly in those growing in arid and nutrient‐deficient environments (Brown et al. 2017). Researchers, both domestically and internationally, have investigated various plant species in arid regions and documented the presence of rhizosheath in the roots of many wild plants (Volkens 1887; Bailey and Scholes 1997). Rhizosheath is especially prevalent in perennial Poaceae species (Duell and Peacock 1985; Ma and Li 2007; Bergmann et al. 2009; Ndour et al. 2020), though they are not limited to these plants and can also be found in some crop species (Brown et al. 2017).

Research on rhizosheath formation mechanisms has intensified in recent years (Marasco et al. 2018). Some studies have suggested that rhizosheath development is more pronounced in herbaceous and shallow‐rooted plants (Brown et al. 2017), and that plants under drought stress tend to form more complete rhizosheath (Iijima et al. 2004). However, species‐specific characteristics and environmental conditions lead to substantial variability in rhizosheath formation and soil‐binding capacity, resulting in notable differences in both the formation and relative mass of rhizosheath across species (Galloway et al. 2022). Thus, depending on environmental factors (like soil moisture content, pH, agglomerate size, porosity, etc.), the ability of different species to integrate with the surrounding soil and form rhizosheath varies. For example, in sandy soils, some species may develop robust rhizosheath, while in other environments, rhizosheath formation may be less significant or absent (Brown et al. 2017; Mo et al. 2022; Tian et al. 2022). This variability introduces uncertainty regarding rhizosheath occurrence among different species.

The rhizosheath is recognized as an important functional trait that enhances plant adaptation to arid environments, exemplifying evolutionary modifications of root systems. Recent studies have highlighted several key herbaceous plant species in the northern Xinjiang desert, such as *Stipagrostis pennata*, *Achnatherum splendens* , and *Agropyron cristatum* , which exhibit rhizosheath formation (Qiu et al. 2012; Luo et al. 2014; Ren et al. 2017). Furthermore, research has shown that some ephemeral annual plant species, which emerge in early spring in the northern Xinjiang desert, also possess rhizosheath. This raises important questions about which species among the annual ephemerals in this region exhibit rhizosheath and what traits are associated with these structures.

This study focuses on annual ephemeral plants in the Gurbantunggut Desert of northern Xinjiang, analyzing variations in rhizosheath formation and characteristics among these species. The goal is to provide a solid theoretical basis for understanding the ecological adaptation strategies employed by annual ephemerals in desert habitats.

## 2 Materials and Methods

### 2.1 Overview of the Study Area

The study was conducted in the northern part of the Xinjiang‐Gurbantunggut Desert, located in the interior of the Junggar Basin, covering an area of approximately 48,800 km^2^. As the second‐largest desert in China and the largest fixed and semi‐fixed desert, it is characterized by a temperate desert climate with distinctive geomorphological features, including extensive stone and gravel surfaces interspersed with scattered sandy areas. The predominant landscape types in this region include sandy and gravelly deserts.

The climate of the Gurbantunggut Desert is marked by extreme temperature fluctuations and limited precipitation. The annual cumulative temperature ranges between 3000 and 3500 degrees centigrade, while annual precipitation is constrained to 70–150 mm. In stark contrast, the annual evaporation rate is notably high, reaching up to 2000 mm. These climatic conditions result in a significant water deficit, particularly during the warmer months. In winter, a stable snow cover forms, with an average thickness of approximately 20 cm. This snow cover is critical as it nearly fully infiltrates the soil, providing essential deep moisture for plant growth in the spring (Wang et al. 2003).

The vegetation of the Gurbantunggut Desert is highly adapted to the harsh arid conditions and consists of a diverse array of desert flora. Early spring ephemeral plants, in particular, represent the main structural component of the herbaceous layer in the ecosystem, contributing to more than 80% of the herbaceous cover during their brief growing season (Zhang and Chen 2002). Notable species within this group include *Malcolmia africana* , *Malcolmia scorpioides*, *Centaurea pulchella*, and *Lappula lasiocarpa* (Wang et al. 2021). These plants play a crucial role in stabilizing the desert ecosystem.

This study focuses on these ephemeral species, which are essential for understanding the ecological dynamics and adaptation strategies of desert plants in response to the region's challenging environmental conditions.

### 2.2 Research Methods

#### 2.2.1 Field Investigation and Sampling

**FIGURE 1:**
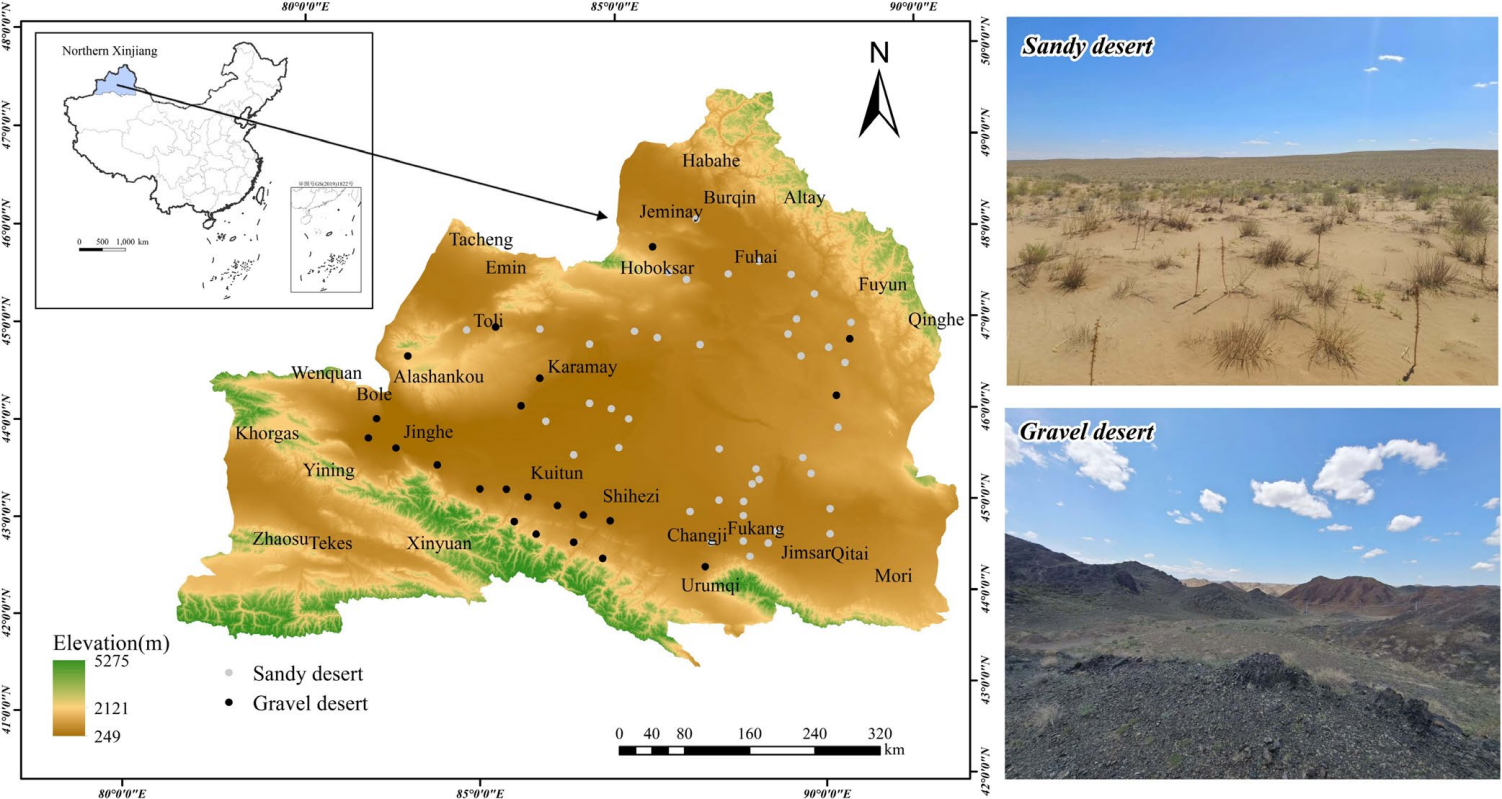
A survey of rhizosheath samples from annual ephemeral plants in two distinct habitat types within the desert region of northern Xinjiang.

Field investigations were conducted in the Gurbantunggut Desert of northern Xinjiang, focusing on areas characterized by sandy and gravelly desert environments with a significant presence of annual ephemeral plant species. The surveys and root sampling took place from early April to late May 2024, corresponding with the active growth period of these plants, which includes both their flowering and fruiting stages. A total of 67 sample points were collected, including 45 sample points in sandy desert and 22 sample points in gravelly desert, the distribution of which is shown in Figure 1. These two habitats are found in different geographic regions of the sandy desert. Sandy deserts have a soil texture dominated by fine sand, while gravelly deserts are characterized by gravel and coarse sand. In each habitat type, we set up multiple sampling sites, ensuring that neighboring sites were spaced more than 10 km apart to avoid spatial autocorrelation. Fifteen plants were collected as biological replicates from each sample site for rhizosheath characterization. In addition, the spacing between neighboring plants within a sample point was maintained at more than 100 m. If a cluster of plants was encountered, only one plant was selected as a sample; this sampling method not only ensured the representativeness of the samples, but also fulfilled the requirement of statistical independence. The primary objective of this investigation was to quantify the number of species exhibiting rhizosheath structures and to analyze the specific characteristics of rhizosheath in these annual ephemeral.

To minimize the impact of competition on root morphology, plants that were not immediately surrounded by other vegetation were selected for sampling. Prior to excavation, carefully plan the path and depth of excavation to avoid unnecessary soil disturbance. Upon identification of a target plant, the entire root system was excavated using a standardized method. A circular area with a radius of 25 cm and a depth of 30 cm around the plant stem was delineated, and the root system was carefully extracted by lifting the soil mass with a shovel. During excavation, try to maneuver carefully to avoid using too much force that may cause damage to the soil structure. Start with the top soil layer and dig down layer by layer. This allows for a better view of the soil structure and reduces disturbance of the underlying soil. Try to ensure the integrity of the root system as much as possible by carefully removing large clods of soil from the root system after digging out the intact plant.

#### 2.2.2 Methods for Assessing Rhizosheath and Scoring Rhizosheath Characteristics

Upon excavation of the plant, the roots were gently shaken to dislodge any loose soil, leaving only the soil adhering tightly to the root system, which formed the rhizosheath. The majority of root segments, apart from the exposed tips, were found to be tightly enmeshed with soil particles, facilitated by root exudates and root hairs, which create a continuous structure through mutual adhesion and entanglement. The rhizosheath structure, which was observed to be approximately 2 mm thick, formed a hollow chamber around the root system, clearly indicating its presence. The assessment and characterization of rhizosheath were carried out following the methodology described by Brown et al. (2017). That is, the presence/absence and strength scores of the rhizosheath were defined by the different adhesion of soil to the roots. These scores range from 0 to 4, with the specific scores being 0 = no soil attached to the root; 1 = only a few grains of soil attached to the root (all soil shaken off); 2 = soil attached to the root (most of the soil will be shaken off); 3 = soil attached to the root remains present after shaking, with most of the soil removed after 5 min in the acoustic bath; and 4 = soil attached to the root stays attached after 5 min of shaking and the acoustic bath. Species with a rhizosheath strength score of ≥ 2 were taken to represent that the rhizosheath was indeed present.

#### 2.2.3 Measurement of Morphological Characteristics of the Rhizosheath

To measure the length and diameter of plant rhizosheaths, all the rhizosheaths of the underground part of each plant were measured using a vernier caliper of the type Guang Lu SF2000 (accuracy 0.01 mm), and the rhizosheath length and maximum diameter were recorded separately. The measurements were repeated for 15 plants of each species, and finally, the average value of each measurement index was taken as the rhizosheath length and diameter of the plant. We selected 15 replicate plants with distinct primary and lateral roots for rhizosheath characterization; single‐rooted plants were excluded from the measurements. Based on these measurements, the surface area and volume of the rhizosheath were calculated using the following formulas: Rhizosheath surface area = π × rhizosheath diameter × rhizosheath length; Rhizosheath volume = π × (rhizosheath diameter/2)^2^ × rhizosheath length;

After digging out the plants, separate the aboveground part from the underground part, shake the underground part to make large pieces of soil fall off, and the rhizosheath soil will remain in the roots. According to the method of Yu et al., use a glass rod to gently press the root system to separate the rhizosheath from the root system, and then use the method of Rabbi et al., carefully brush off the rhizosheath soil with a soft brush and collect it to ensure that the root system and root hairs are not damaged in the process. After rhizosheath is separated from the root system, put it into an oven (75°C, 72 h) and bake it to a constant mass, then use sartorius bs210s electronic balance (0.0001 g) to weigh the rhizosheath (Rabbi et al. 2018; Yu et al. 2021). The relative weight of the rhizosheath was calculated using the following equation: Rhizosheath relative weight (g/cm) = dry weight of rhizosheath soil (g)/total root length (cm). Additionally, the rhizosheath development index was calculated as the ratio of the dry mass of the rhizosheath soil to the mass of the roots (Amellal et al. 1998).

Among them, after the rhizosheath is detached from the root system and the rhizosheath intensity is scored, the impurities on the root surface are removed with distilled water, and the damage to root hairs is minimized during the cleaning process. Spread the cleaned root system horizontally, and try to make the root system extend without crossing, so as to facilitate subsequent measurement. Epson Expression 13000XL root scanner (Epson, Los Alamitos, CA USA) was used to scan the root system of each plant, and the obtained root scanning map was numbered and saved. Import the scanned image file into WinRhizo STD4800 LA2400 root analysis software for analysis, and obtain the parameter of total root length. Put the scanned clean roots into the oven (75°C, 72 h) and bake them to a constant mass. Then weigh the rhizosheath with Sartorius BS210s electronic balance (0.0001 g) to obtain the parameter of root dry weight.

#### 2.2.4 Data Processing and Analysis

Excel was used for data collation of species types and numbers, and SPSS 19.0 software was used for one‐way ANOVA (One‐way ANOVA) to analyze the variability of rhizosheath characteristics among species. All rhizosheath morphology data had to satisfy normal distribution and chi‐squaredness to comply with one‐way ANOVA (One‐way ANOVA) before analysis. If the data did not satisfy normal distribution or chi‐square, a transformation of the data (logarithmic or square root) was required, and the significance level of the test was *p* = 0.05. Finally, the tabulation and plotting were completed using Origin 2022 and ArcGIS 10.7 (ESRI).

## 3 Results

### 3.1 Characteristics of the Rhizosheath of Annual Ephemerals in the Deserts of Northern Xinjiang

#### 3.1.1 Characteristics of Rhizosheath in Annual Ephemerals

Field investigations and systematic specimen identification revealed that a total of 67 species of annual ephemeral plants were sampled within the sandy and gravelly habitats of the northern Xinjiang desert (Table 1). These species were distributed across 14 families and 48 genera. Among the families represented, the Cruciferae (Brassicaceae) family was the most dominant, with 27 species, accounting for 40% of the total species investigated. The Asteraceae family followed, contributing 15% of the total species. In contrast, several families—Euphorbiaceae, Geraniaceae, Primulaceae, Lamiaceae, Ranunculaceae, and Solanaceae—were represented by only one species each, collectively constituting just 1.49% of the total species identified.

**TABLE 1:** A questionnaire on the presence of rhizosheath of annual ephemeral species in the deserts of northern Xinjiang.

Species	Family	Genus	Rhizosheath or not		Rhizosheath score
			Sandy deserts	Gravel desert	(0–4) Sandy/gravel
*Descurainia sophia*	Brassicaceae	*Descurainia*	**√**	**×**	2/0
*Alyssum dasycarpum*	Brassicaceae	*Alyssum*	**×**	**×**	
*Meniocus linifolius*	Brassicaceae	*Meniocus*	**×**	—	
*Leptaleum filifolium*	Brassicaceae	*Leptaleum*	**×**	—	
*Camelina microcarpa*	Brassicaceae	*Camelina*	—	**×**	
*Chorispora sibirica*	Brassicaceae	*Chorispora*	—	**×**	
*Chorispora tenella*	Brassicaceae	*Chorispora*	○	**×**	
*Diptychocarpus strictus*	Brassicaceae	*Diptychocarpus*	—	**×**	
*Strigosella africana*	Brassicaceae	*Strigosella*	**×**	—	
*Strigosella scorpioides*	Brassicaceae	*Strigosella*	**×**	○	
Sisymbrium altissimum	Brassicaceae	*Sisymbrium*	—	**×**	
*Tetracme quadricornis*	Brassicaceae	*Tetracme*	—	**×**	
*Tetracme recurvata*	Brassicaceae	*Tetracme*	**×**	—	
*Rudolf‐kamelinia korolkowii*	Brassicaceae	*Rudolf‐kamelinia*	○	**×**	
*Lepidium perfoliatum*	Brassicaceae	*Lepidium*	**×**	○	
*Lepidium ruderale*	Brassicaceae	*Lepidium*	**×**	—	
*Lepidium chalepense*	Brassicaceae	*Lepidium*	—	**×**	
*Thlaspi arvense*	Brassicaceae	*Thlaspi*	—	**×**	
Capsella bursa‐pastoris	Brassicaceae	*Capsella*	—	**×**	
*Euclidium syriacum*	Brassicaceae	*Euclidium*	—	**×**	
*Goldbachia laevigata*	Brassicaceae	*Goldbachia*	**×**	○	
*Goldbachia sabulosa*	Brassicaceae	*Goldbachia*	**×**	○	
*Lachnoloma lehmannii*	Brassicaceae	*Lachnoloma*	**×**	○	
*Isatis multicaulis*	Brassicaceae	*Isatis*	—	**×**	
*Isatis gymnocarpa*	Brassicaceae	*Isatis*	—	**×**	
*Isatis violascens*	Brassicaceae	*Isatis*	**×**	—	
*Isatis minima*	Brassicaceae	*Isatis*	**×**	—	
*Heteracia szovitsii*	Asteraceae	*Heteracia*	**×**	—	
*Centaurea pulchella*	Asteraceae	*Centaurea*	**√**	○	2/—
*Crepis desertorum*	Asteraceae	*Crepis*	**×**	—	
*Epilasia hemilasia*	Asteraceae	*Epilasia*	**×**	○	
*Koelpinia linearis*	Asteraceae	*Koelpinia*	**×**	—	
*Amberboa turanica*	Asteraceae	*Amberboa*	—	**×**	
*Lactuca undulate*	Asteraceae	*Lactuca*	**√**	**×**	2/0
*Senecio subdentatus*	Asteraceae	*Senecio*	**×**	○	
*Echinops gmelinii*	Asteraceae	*Echinops*	**×**	○	
*Filago arvensis*	Asteraceae	*Filago*	○	**×**	
*Lappula lasiocarpa*	Boraginaceae	*Lappula*	**×**	**—**	
*Lappula semiglabra*	Boraginaceae	*Lappula*	○	**√**	—/2
*Lappula macra*	Boraginaceae	*Lappula*	**×**	—	
*Lappula spinocarpos*	Boraginaceae	*Lappula*	**×**	○	
*Rochelia bungei*	Boraginaceae	*Rochelia*	**×**	○	
*Arnebia decumbens*	Boraginaceae	*Arnebia*	**×**	—	
*Nonea caspica*	Boraginaceae	*Nonea*	**√**	○	2/—
*Eremopyrum distans*	Poaceae	*Eremopyrum*	**√**	**√**	4/4
*Eremopyrum bonaepartis*	Poaceae	*Eremopyrum*	○	**√**	—/2
*Eremopyrum orientale*	Poaceae	*Eremopyrum*	**√**	**√**	3/4
*Eremopyrum triticeum*	Poaceae	*Eremopyrum*	**√**	**√**	4/2
*Schismus arabicus*	Poaceae	*Schismus*	**√**	—	2/—
*Bromus tectorum*	Poaceae	*Bromus*	—	**√**	—/2
*Astragalus arpilobus*	Leguminosae	*Astragalus*	**×**	○	
*Astragalus oxyglottis*	Leguminosae	*Astragalus*	○	**×**	
*Astragalus persepolitanus*	Leguminosae	*Astragalus*	**×**	—	
*Trigonella arcuata*	Leguminosae	*Trigonella*	**√**	○	3/—
*Trigonella cancellata*	Leguminosae	*Trigonella*	○	**×**	
*Hypecoum erectum*	Papaveraceae	*Hypecoum*	**×**	—	
*Hypecoum parviflorum*	Papaveraceae	*Hypecoum*	**×**	○	
Euphorbia turczaninowii	Euphorbiaceae	*Euphorbia*	**×**	○	
*Erodium oxyrhinchum*	Geraniaceae	*Erodium*	**×**	○	
*Androsace maxima*	Primulaceae	*Androsace*	**×**	**√**	0/2
*Veronica rubrifolia*	Plantaginaceae	*Veronica*	**×**	○	
*Plantago minuta*	Plantaginaceae	*Plantago*	**×**	○	
*Nepeta micrantha*	Lamiaceae	*Nepeta*	**√**	○	2/—
*Atriplex dimorphostegia*	Amaranthaceae	*Atriplex*	**×**	—	
*Corispermum lehmannianum*	Amaranthaceae	*Corispermum*	**×**	—	
*Ceratocephala testiculata*	Ranunculaceae	*Ceratocephala*	○	**×**	
*Hyoscyamus pusillus*	Solanaceae	*Hyoscyamus*	**√**	**×**	2/0

*Note:* Species names and family names refer to the Plantwise (http://www.iplant.cn/) website using the APGIV classification system. “√” indicates that the species was present in the rhizosheath during the survey, “×” indicates that the species was not present in the rhizosheath during the survey, “○” indicates that the species was distributed in this habitat but was not collected and the presence of rhizosheath is unknown; ‘−’ indicates the species has no distribution in this habitat.

**FIGURE 2:**
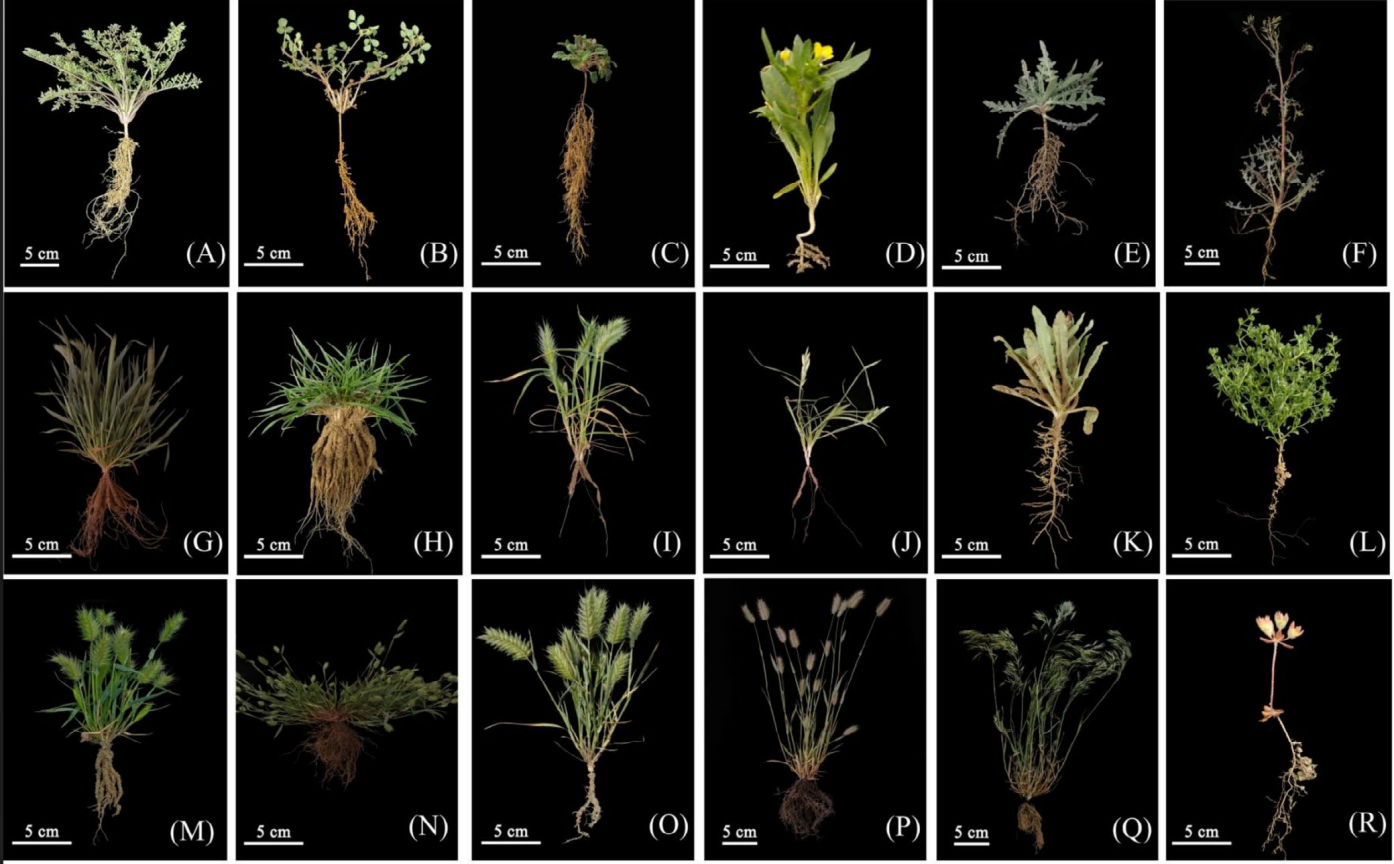
Photographs of morphological features of rhizosheath of annual ephemerals with rhizosheath. (A–K) sandy soil, (L–R) gravel soil; (A) *Descurainia sophia* ; (B) *Trigonella arcuat*; (C) *Nepeta micrantha*; (D) *Hyoscyamus pusillus*; (E) *Centaurea pulchella*; (F) *Lactuca undulata*; (G) *Eremopyrum distans*; (H) *Eremopyrum triticeum* ; (I) *Eremopyrum orientale* ; (J) *Schismus arabicus* ; (K) *Nonea caspica*; (L) *Lappula semiglabra*; (M) *Eremopyrum distans*; (N) *Eremopyrum triticeum* ; (O) *Eremopyrum orientale* ; (P) *Eremopyrum bonaepartis* ; (Q) *Bromus tectorum* ; (R) *Androsace maxima* .

**FIGURE 3:**
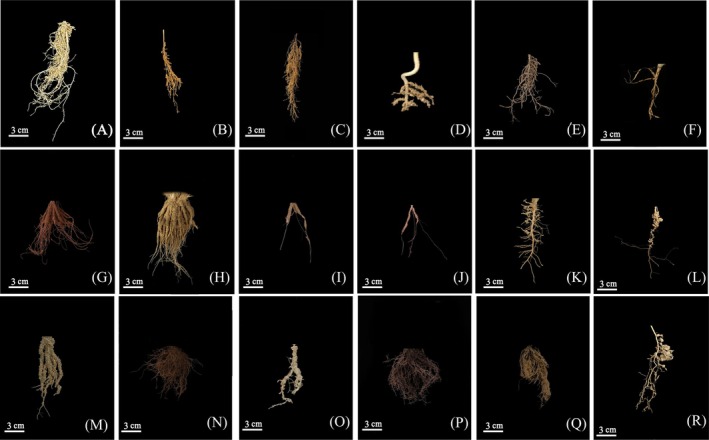
Detailed view of rhizosheath morphology of plants with rhizosheath in annual ephemerals. (A −K) sandy soil, (L −R) gravel soil (A) *Descurainia sophia* ; (B) *Trigonella arcuat*; (C) *Nepeta micrantha*; (D) *Hyoscyamus pusillus*; (E) *Centaurea pulchella*; (F) *Lactuca undulata*; (G) *Eremopyrum distans*; (H) *Eremopyrum triticeum* ; (I) *Eremopyrum orientale* ; (J) *Schismus arabicus* ; (K) *Nonea caspica*; (L) *Lappula semiglabra*; (M) *Eremopyrum distans*; (N) *Eremopyrum triticeum* ; (O) *Eremopyrum orientale* ; (P) *Eremopyrum bonaepartis* ; (Q) *Bromus tectorum* ; (R) *Androsace maxima* .

Statistical analysis of the 67 species revealed that 15 species exhibited the presence of a rhizosheath structure (Figures 2 and 3), representing 8 families and 12 genera. In contrast, 52 species were found to lack rhizosheath structures, spanning 10 families and 38 genera. Among the species with rhizosheath, the Poaceae family was the most prevalent, contributing six species, or 40% of all rhizosheath‐forming species. At the genus level, *Eremopyrum* was the most dominant, with four species exhibiting rhizosheath structures, accounting for 27% of all rhizosheath species.

These findings highlight the diverse distribution of rhizosheath among annual ephemeral plants in the region, with certain families and genera showing a higher propensity for rhizosheath formation. This variability underscores the ecological significance of rhizosheath as an adaptive trait, particularly within specific taxonomic groups such as Poaceae.

#### 3.1.2 Characteristics of the Rhizosheath of Annual Ephemerals in Various Habitats

**FIGURE 4:**
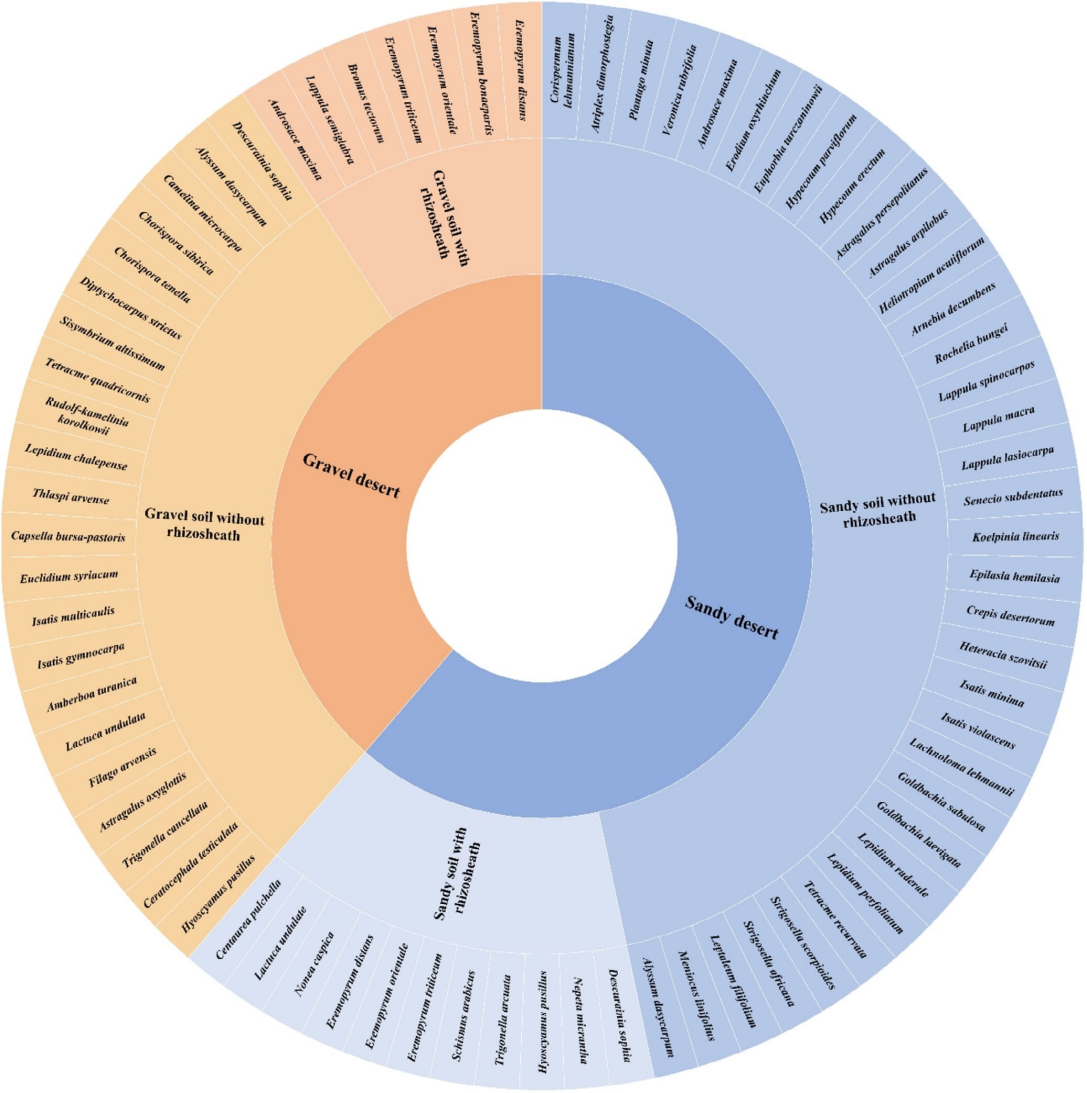
Presents the species statistics of annual ephemerals, categorized by the presence or absence of rhizosheath, across two distinct habitats.

The survey results show that annual ephemeral plants are distributed across both sandy and gravelly habitats in the northern Xinjiang desert, encompassing a total of 41 genera and 60 species. Specifically, 33 genera and 39 species were found in sandy habitats, while 20 genera and 21 species were identified in gravelly habitats (Figure 4). Additionally, 7 genera and 9 species were observed in both habitat types. Sandy habitats exhibited a greater abundance of annual ephemerals, accounting for 58.2% of the total species observed.

Regarding rhizosheath formation, a total of 12 genera and 12 species were found to exhibit rhizosheath across these habitats. In sandy habitats, 11 species with rhizosheath structures were identified. These included *Descurainia sophia* (Brassicaceae), *Centaurea pulchella* and *Lactuca undulata* (Asteraceae), *Nonea caspica* (Boraginaceae), *Eremopyrum distans*, *Eremopyrum orientale* , *Eremopyrum triticeum* , and *Schismus arabicus* (Poaceae), *Nepeta micrantha* (Lamiaceae), *Hyoscyamus pusillus* (Solanaceae), and *Trigonella arcuata* (Leguminosae).

In gravelly habitats, seven species with rhizosheath structures were recorded, including *Eremopyrum bonaepartis* , *Eremopyrum distans*, *Eremopyrum orientale* , *Eremopyrum triticeum* (Poaceae), *Lappula semiglabra* (Boraginaceae), and *Androsace maxima* (Primulaceae). Notably, three species—*Eremopyrum distans*, *Eremopyrum orientale* , and *Eremopyrum triticeum* —exhibited rhizosheath structures in both sandy and gravelly habitats.

These findings highlight the adaptability of certain species, particularly within the genus *Eremopyrum*, which demonstrate rhizosheath formation across different habitat types. The greater abundance of rhizosheath‐forming species in sandy habitats underscores the importance of soil texture and environmental conditions in promoting rhizosheath development.

### 3.2 Variations in the Characteristics of Rhizosheath Formation Among Different Species of Annual Ephemerals

#### 3.2.1 Variations in Rhizosheath Formation Characteristics Among Different Species

Analyses of rhizosheath formation in the 15 species that exhibited rhizosheath structures revealed considerable interspecific variability, as shown in Figure 2 and detailed in Table 2. These differences were primarily observed in the morphology of the rhizosheath, including their shape, size, surface area, and other structural attributes, as well as in their overall weight. The rhizosheath generally appeared in cylindrical or conical shapes, though in some species, they formed discontinuous, ball‐like structures along the roots.

**TABLE 2:** Comparison of rhizosheath formation characteristics among different species.

Species	Rhizosheath length (mm)	Rhizosheath diameter (mm)	Rhizosheath surface area (mm^2^)	Rhizosheath volume (mm^3^)	Rhizosheath relative weight (g/cm)	Rhizosheath development index
*Eremopyrum bonaepartis*	123.31 ± 10.98^a^	1.24 ± 0.53^e^	474.37 ± 190.57^de^	169.92 ± 133.82^cd^	0.03 ± 0.01^cd^	79.93 ± 102.03^cd^
*Eremopyrum distans*	113.20 ± 4.79^ab^	2.61 ± 0.69^bcd^	954.03 ± 281.2^a^	751.30 ± 480.79^ab^	0.04 ± 0.03^bcd^	74.75 ± 60.79^bcd^
*Eremopyrum orientale*	81.52 ± 14.54^d^	2.78 ± 0.97^abc^	828.03 ± 412.81^ab^	811.72 ± 626.96^a^	0.06 ± 0.02^bc^	41.45 ± 13.23^bc^
*Eremopyrum triticeum*	91.66 ± 9.75^cd^	2.08 ± 0.42^cde^	696.33 ± 165.65^bc^	463.44 ± 213.33^bc^	0.11 ± 0.07^a^	37.83 ± 14.96^a^
*Bromus tectorum*	104.15 ± 17.91^bc^	1.62 ± 0.41^e^	526.40 ± 145.66^cd^	224.41 ± 108.70^cd^	0.03 ± 0.02^cd^	39.22 ± 28.48^cd^
*Schismus arabicus*	35.46 ± 13.22^gh^	1.92 ± 0.68^cde^	201.75 ± 77.01^g^	101.92 ± 59.67^d^	0.03 ± 0.01^cd^	69.52 ± 66.32^cd^
*Descurainia sophia*	46.50 ± 8.90^fg^	1.71 ± 0.37^de^	247.49 ± 61.75^fg^	109.19 ± 41.83^d^	0.03 ± 0.02^cd^	21.78 ± 38.63^cd^
*Centaurea pulchella*	33.82 ± 5.08^gh^	3.61 ± 0.91^a^	385.25 ± 119.64^defg^	370.11 ± 202.10^cd^	0.08 ± 0.05^ab^	45.88 ± 14.89^ab^
*Lactuca undulate*	45.88 ± 10.23^fg^	3.14 ± 1.16^ab^	438.07 ± 168.77^def^	379.76 ± 252.66^cd^	0.05 ± 0.06^bcd^	27.60 ± 28.21^bcd^
*Nonea caspica*	32.27 ± 6.02^hi^	2.63 ± 0.87^bcd^	262.52 ± 96.29^e^ ^fg^	188.95 ± 130.70^cd^	0.01 ± 0.02^d^	5.16 ± 4.44^d^
*Lappula semiglabra*	20.28 ± 3.99^i^	2.81 ± 0.85^abc^	180.70 ± 71.74^g^	138.89 ± 90.09^d^	0.03 ± 0.02^bcd^	6.03 ± 4.42^bcd^
*Trigonella arcuata*	38.45 ± 10.65^gh^	2.59 ± 0.84^bcd^	321.55 ± 175.43^defg^	236.98 ± 216.81^cd^	0.08 ± 0.03^ab^	14.87 ± 7.45^ab^
*Nepeta micrantha*	62.12 ± 14.39^e^	1.84 ± 0.82^de^	351.81 ± 168.89^defg^	190.23 ± 179.49^cd^	0.02 ± 0.02^cd^	12.17 ± 10.71^cd^
*Androsace maxima*	55.88 ± 10.84^ef^	1.61 ± 0.12^e^	281.04 ± 54.64^efg^	113.05 ± 24.06^d^	0.02 ± 0.01^d^	15.48 ± 8.27^d^
*Hyoscyamus pusillus*	26.69 ± 6.56^hi^	3.21 ± 0.81^ab^	261.57 ± 63.78^efg^	215.98 ± 93.61^cd^	0.06 ± 0.07^bc^	3.16 ± 2.56^b^ ^c^

*Note:* Data are presented as mean ± standard deviation, and different lowercase letters after data in the same column indicate significant differences (*p* < 0.05), as in the table below.

Rhizosheath were predominantly concentrated in the middle and upper portions of the root system, with a notable density in the lateral roots, particularly in regions associated with root hairs. However, no rhizosheath formation was observed in the apical (root tip) regions. The length of rhizosheath varied significantly across species, ranging from 20.3 to 123.31 mm. In terms of weight, the relative weight of rhizosheath also varied widely, from 0.01 to 0.11 g/cm, indicating substantial differences in rhizosheath development between species.

The rhizosheath development indices, which quantify the mass of the rhizosheath relative to the root mass, also showed significant variability, further highlighting the diversity in rhizosheath formation among different species. These findings underscore the adaptive strategies of different annual ephemeral species in forming rhizosheath, which likely play a critical role in their survival and ecological success in arid desert environments.

#### 3.2.2 Variations in the Characteristics of Rhizosheath Formation Across Diverse Habitats

The data summarized in Table 3 demonstrate significant differences in the characteristics of rhizosheath formation between sandy and gravelly habitats. In sandy habitats, the rhizosheath diameter, surface area, volume, and development index of annual ephemeral plants were higher than those in gravelly habitats, with increases of 42.32%, 47.65%, 133.07%, and 31.53%, respectively. These findings suggest that sandy environments provide more favorable conditions for the formation of thicker, denser rhizosheath, potentially due to better soil aggregation facilitated by fine sand particles.

**TABLE 3:** Comparison of the characteristics of rhizosheath formation in annual ephemerals in two habitats.

Habitats	Rhizosheath length (mm)	Rhizosheath diameter (mm)	Rhizosheath surface area (mm^2^)	Rhizosheath volume (mm^3^)	Rhizosheath relative weight (g/cm)	Rhizosheath development index
Sandy habitat	64.92 ± 12.83^a^	2.69 ± 0.18^a^	564.72 ± 127.6^a^	463.63 ± 124.29^a^	0.06 ± 0.01^a^	39.75 ± 10.08^a^
Gravelly habitat	69.07 ± 12.98^a^	1.89 ± 0.19^b^	382.48 ± 60.56^b^	198.92 ± 38.8^b^	0.03 ± 0^a^	30.22 ± 8.7^a^

*Note:* Data are presented as mean ± standard error. Different lowercase letters within the same column indicate significant differences (*p* < 0.05).

In contrast, the rhizosheath length was notably greater in gravelly habitats, with increases of 6.3%, respectively. This indicates that, while gravelly soils may not support as dense or compact rhizosheath as sandy soils, they promote the formation of longer and more extensive rhizosheath structures. These differences may reflect adaptations to the distinct soil textures and moisture dynamics of each habitat, with plants in gravelly environments possibly relying on longer rhizosheath to maximize root‐soil contact for water and nutrient uptake.

Overall, these results highlight the influence of habitat type on rhizosheath morphology and development, with different environmental conditions promoting distinct patterns of rhizosheath formation in annual ephemeral plants.

**FIGURE 5:**
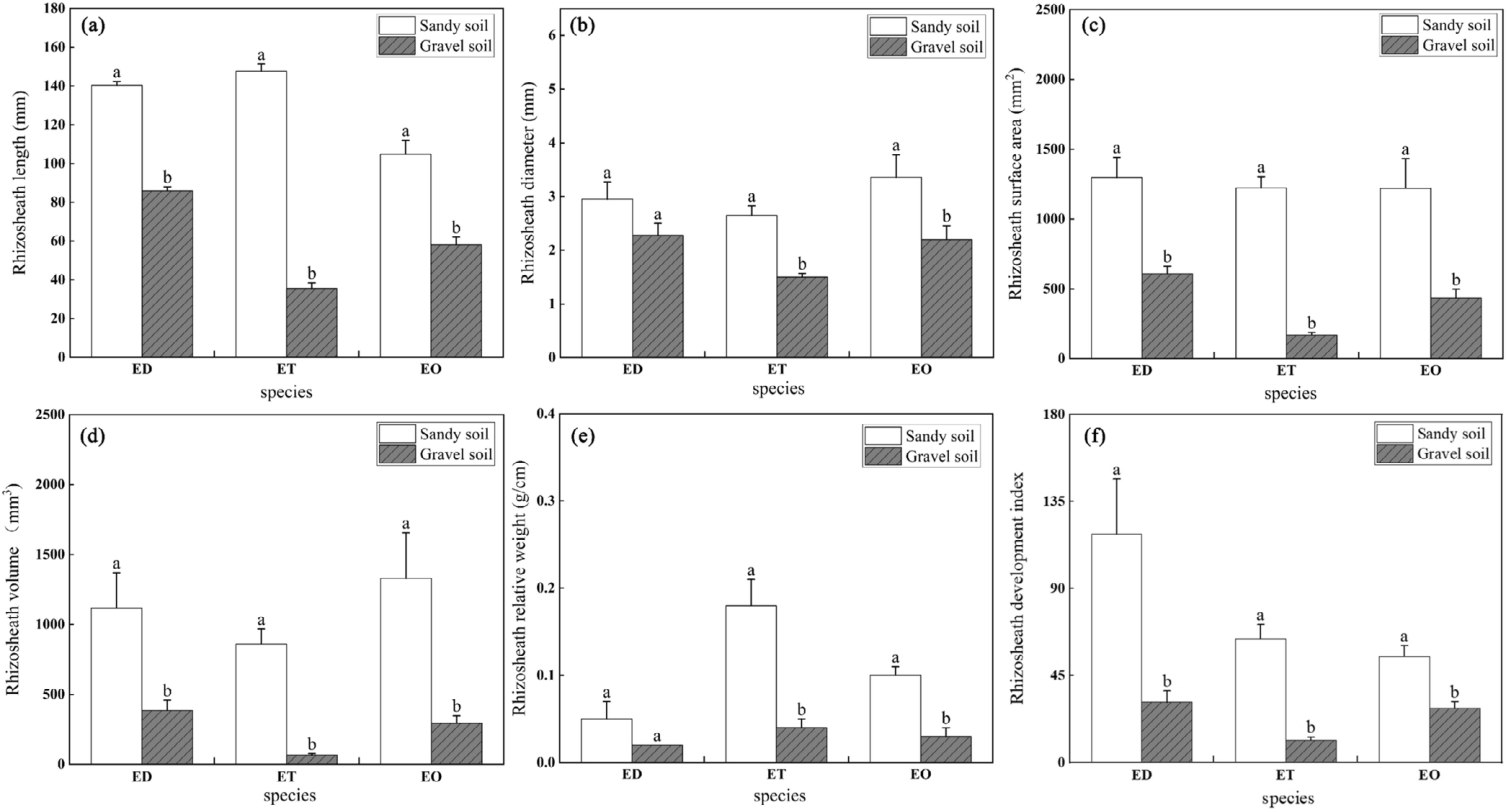
Comparison of rhizosheath formation characteristics of the three plants in two habitats, sandy and gravelly. Different lower case letters indicate significant differences (*p* < 0.05). ED, *Eremopyrum distans*；ET, *Eremopyrum triticeum* ；EO, *Eremopyrum orientale* ; (a) Rhizosheath length; (b) Rhizosheath diameter; (c) Rhizosheath surface area; (d) Rhizosheath volume; (e) Rhizosheath relative weight; (f) Rhizosheath development index.

Species that exhibited rhizosheath structures in both sandy and gravelly habitats included *Eremopyrum distans*, *Eremopyrum orientale* , and *Eremopyrum triticeum* . Across all three species, the relative weight, surface area, volume, and development index of the rhizosheath were consistently greater in sandy habitats compared to gravelly habitats. Notably, the rhizosheath surface area and volume in sandy habitats were significantly higher (*p* < 0.05) than in gravelly habitats (Figure 5).

For *Eremopyrum distans*, the rhizosheath surface area and volume in sandy habitats were 2.13 and 2.90 times larger, respectively, than those in gravelly habitats (*p* < 0.05). Similarly, *Eremopyrum triticeum* displayed significantly higher rhizosheath length, relative weight, and development index in sandy habitats, with increases of 4.14, 4.5, and 5.52 times, respectively, compared to gravelly habitats (*p* < 0.05). In the case of *Eremopyrum orientale* , the rhizosheath surface area and volume in sandy habitats exceeded those in gravelly habitats by factors of 2.80 and 4.52, respectively (*p* < 0.05).

These results highlight the marked influence of habitat type on rhizosheath development, with sandy environments favoring the formation of larger, more extensive rhizosheath across these species. The increased rhizosheath size in sandy habitats suggests that soil texture plays a crucial role in facilitating the development of this adaptive structure, which enhances root‐soil interactions for improved water and nutrient acquisition.

## 4 Discussion

### 4.1 Characteristics and Variability of Rhizosheath in Annual Ephemerals

The presence and variability of rhizosheath in different plant species are likely influenced by both genetic factors and environmental conditions. Previous studies, including those by Volkens (1887), Bailey and Scholes (1997), and Smith et al. (2011), have documented the widespread occurrence of rhizosheath across various angiosperm families, such as Gramineae, Leguminosae, and Labiatae. This study aligns with these findings, as we observed rhizosheath structures in 15 of the 70 species examined in the Gurbantunggut Desert, with the majority belonging to the Poaceae family, representing 40% of the total rhizosheath‐forming species. These results support earlier research by Duell and Peacock (1985), Ma and Li (2007), Bergmann et al. (2009), and Ndour et al. (2020), which suggested that rhizosheath formation is more common in Gramineae species, particularly in arid and nutrient‐deficient environments.

The interspecific variability in rhizosheath morphology observed in this study (Result 3.2.1) can be attributed to differences in root system structure and root hair development. Root morphology, including the twisting, elongation, and infiltration of roots into the soil, has been shown to influence the soil particles adsorbed by the roots, affecting rhizosheath formation (North and Nobel 1997). The number of lateral roots and root hairs also plays a critical role, as an increased number of lateral roots can enhance soil particle aggregation, leading to more extensive rhizosheath formation (Ndour et al. 2020). Additionally, root hairs increase the root–soil contact area, facilitating greater soil particle adhesion and larger rhizosheath (Rongsawat et al. 2021). Liu et al. (2019) further demonstrated that in *Setaria italica* , rhizosheath size and weight were positively correlated with root hair density under dry soil conditions.

Phylogenetic studies, such as those by Smith et al. (2011), have suggested that rhizosheath formation may be an ancestral trait within certain plant families, showing phylogenetic conservation. However, there is still limited research on the prevalence of rhizosheath across the angiosperm phylogeny. This study highlights the need for further investigations to determine the extent to which plant genetic factors contribute to rhizosheath formation, as the current understanding remains incomplete.

### 4.2 Rhizosheath Characteristics Across Various Habitats and Their Variability

The morphological diversity of rhizosheath across species and habitats reflects the adaptive strategies employed by plants to thrive in different ecological environments. Larger rhizosheath, for example, increases the contact area between roots and soil, enhancing water and nutrient uptake. In contrast, smaller rhizosheath may represent a resource‐saving strategy, minimizing root biomass investment in nutrient‐poor or arid environments. Annual ephemeral plants, which rely on brief periods of moisture from early spring rains and snow, benefit from rhizosheath by enhancing their ability to absorb water and nutrients during their rapid growth cycle (Mao and Zhang 1994). This adaptation allows them to complete their life cycle before the onset of summer drought, highlighting the critical role of rhizosheath in desert ecosystems.

Our study found that rhizosheath formation was more prevalent in sandy habitats compared to gravelly habitats, likely due to differences in soil aggregate composition. Sandy soils contain smaller particles (between 0.25 and 2 mm), which are more easily encapsulated by root and microbial secretions, promoting rhizosheath formation (Luo et al. 2014). In contrast, gravelly soils, which consist of larger particles, are less conducive to rhizosheath formation. Soil moisture also plays a significant role, as drier, sandier soils are associated with larger rhizosheath, likely due to increased root hair development in response to low water availability (Haling et al. 2010; Aslam et al. 2022; Zhang et al. 2023). Microorganisms, such as those found in the rhizosphere of barley, have been shown to promote rhizosheath formation through the secretion of growth hormones, further enhancing the role of sandy habitats in fostering rhizosheath (Xu et al. 2023). A comparison of three *Eremopyrum* species with rhizosheath across sandy and gravelly habitats revealed that rhizosheath traits, such as length, diameter, weight, surface area, volume, and development index, were consistently greater in sandy habitats. Sandy soils, with lower porosity and greater root‐soil contact, promote the formation of larger rhizosheath, increasing root efficiency in nutrient absorption (Hallett et al. 2022). Additionally, sandy soils generally have lower nutrient content compared to gravelly soils, which contain higher levels of organic matter, total nitrogen, and phosphorus (*p* < 0.01). This suggests that rhizosheath may act as nutrient retainers in resource‐poor environments, enhancing plant access to growth nutrients, especially under low nitrogen conditions, where rhizosheath‐associated bacteria symbiotically assist plant growth (Cortivo et al. 2017).

In summary, this study demonstrates that rhizosheath formation varies significantly among species and habitats, with sandy soils providing more favorable conditions for the development of rhizosheath. Although research on rhizosheath formation has grown in recent years, a comprehensive understanding of the factors driving its formation remains elusive. It is unclear whether genetic factors, environmental influences, or a combination of both dominates rhizosheath development. To further clarify the mechanisms behind rhizosheath formation, future studies should expand research to include a broader range of ecosystems and species. Additionally, investigations into the genetic regulation of root systems and the environmental factors influencing rhizosheath growth and development are necessary to deepen our understanding of this critical plant adaptation.

## 5 Conclusion

Rhizosheath formation in short‐lived annual ephemeral plants exhibits significant interspecific variation. Among the 67 species surveyed in the Gurbantunggut Desert, representing 14 families and 48 genera, 15 species were found to possess distinct rhizosheath structures. The majority of these species belonged to the Poaceae family, which accounted for approximately 40% of the total rhizosheath‐forming species. Rhizosheath formation results from complex interactions between plants, soil, and microbes, and its occurrence varies across different ecological environments.

In terms of habitat, 11 species with rhizosheath were identified in sandy habitats, while 7 species were found in gravelly habitats. The rhizosheath characteristics, including length, diameter, weight, surface area, volume, and development index, were consistently larger in sandy habitats than in gravelly habitats for species such as *Eremopyrum distans*, *Eremopyrum triticeum* , and *Eremopyrum orientale* . These findings indicate that sandy environments provide more favorable conditions for rhizosheath formation in annual ephemerals.

Despite the importance of rhizosheath as a root trait that influences plant growth, the underlying causes of its variability across species and habitats remain unclear. Whether this variability is primarily driven by species‐specific genetic factors, external environmental conditions, or microbial activity in the rhizosphere warrants further investigation. Future research should focus on elucidating the mechanisms behind rhizosheath formation and its ecological significance in different plant species and habitats.

## Author Contributions

**Yu Ding:** data curation (equal), investigation (supporting), validation (equal), visualization (equal), writing – original draft (supporting), writing – review and editing (equal). **Cheng Lv:** data curation (equal), investigation (equal), validation (equal). **Kangwei Jiang:** data curation (equal), investigation (equal). **Xinyu Xia:** data curation (equal). **Zhiqing Zhang:** data curation (equal). **Qingqing Zhang:** conceptualization (equal), funding acquisition (equal), methodology (equal), project administration (equal), supervision (equal).

## Ethics Statement

No specific permits were required for the described field studies. The locations are not privately owned or protected in any way, and the field studies did not involve endangered or protected species.

## Conflicts of Interest

The authors declare no conflicts of interest.

## Data Availability

The original contributions presented in the study are included in the article; further inquiries can be directed to the corresponding author.
